# Clinician’s Perceptions of a CME Activity to Increase Knowledge of Vaccination in Adults with Chronic Inflammatory Conditions

**DOI:** 10.35248/2684-1630.19.4.146

**Published:** 2019-11-29

**Authors:** Saira Z Sheikh, Edward G A Iglesia, Matthew Underwood, Shruti Saxena-Beem, Mildred Kwan

**Affiliations:** 1 University of North Carolina Thurston Arthritis Research Center, Chapel Hill, North Carolina, USA; 2 Department of Medicine, Division of Rheumatology, Allergy and Immunology, University of North Carolina at Chapel Hill, North Carolina, USA; 3 School of Medicine, University of North Carolina, Chapel Hill, North Carolina, USA

**Keywords:** Autoimmune diseases, Continuing medical education, SLE, Systemic lupus erythematosus, Immunocompromised host, Influenza vaccines, Pneumococcal vaccines, Rheumatic diseases

## Abstract

**Objective:**

Annual influenza and pneumococcal vaccination rates remain suboptimal in patients with systemic lupus erythematosus despite their higher risk of infections and related complications. The CDC identified lack of knowledge about vaccine guidelines among adult patients and their providers as the most substantial barrier to vaccination coverage. As specialists working with particularly affected populations, rheumatologists, allergists, and immunologists can advise patients regarding gaps in recommended vaccinations.

The aim of this study was to describe prescribers’ perceptions of an educational activity that was developed to increase rates of appropriate pneumococcal and influenza vaccination in adults with chronic inflammatory conditions. We were interested in the impact of the educational activity on the knowledge and practice of providers.

**Methods:**

We evaluated a multimodal educational activity aimed at increasing vaccination rates in high-risk adults. We assessed provider knowledge, perceptions of the activity, and impact on their practice. The activity was conducted at a single site “in house” education event in the live format and was disseminated nationally in print and online format.

**Results:**

In the “in house” interactive education session, mean scores on the pre- and post-tests were 75% (SD 11.6%, 95% CI 70–80%) and 89% (SD 11.1%, 95% CI 85–95%; p=.0001 vs. pre-test score), respectively, demonstrating that knowledge was significantly increased after completing the activity. In the nationally available activity 93% (n=240) of respondents indicated that the activity significantly increased their awareness about the importance of vaccinations in these high-risk patients and recognition of when these vaccines were indicated or contraindicated, while 55% (n=142) planned to consequently change their practice.

**Conclusion:**

Provider education is a valuable strategy for practice-based improvements in vaccination coverage since provider failure to recommend vaccinations is a primary barrier in high-risk patients. Most patients received vaccinations based on physician recommendations and vaccination rates were markedly higher among patients receiving vaccine information from their providers. This educational activity increased clinicians’ knowledge of and confidence in vaccinations for adults with chronic inflammatory conditions.

## INTRODUCTION

Patients with systemic lupus erythematosus (SLE), a chronic inflammatory disease, are at higher risk for infections compared to the general population, and complications from these infections are more frequent in this patient population [1–3]. Patients with SLE have a 13-fold higher risk of developing invasive pneumococcal infection compared with general population [4]. The increased risk of infection in chronic inflammatory disease is attributable both to an altered immune response associated with the immune condition itself and to the immunosuppressive treatments required to control the underlying inflammatory condition [5].

This greater susceptibility to infection is precisely why increasing vaccine coverage in lupus patients is vital since respiratory infections from Streptococcus pneumoniae and other agents are the leading cause of serious infections in SLE [6,7] and infections still account for approximately one-third of SLE deaths despite a decreasing trend of mortality in these patients [8]

Patients with other chronic inflammatory diseases also display this higher risk for infections and more frequent complications seen in SLE patients. For example, patients with rheumatoid arthritis (RA) have a 2.75-fold increase in incidence of influenza complications compared to controls, and between 7.1 and 33.3 times the rate of invasive pneumococcal disease [3–9] In patients with asthma, a chronic inflammatory respiratory disease, influenza infection is associated with more severe and frequent viral lower respiratory tract infections, pneumonia, and secondary bacterial infections [10]

Despite the high risk of infections and complications, strong evidence of vaccine safety and efficacy, and recommendations from national organizations, vaccination rates remain suboptimal with 40% of SLE patients remaining unvaccinated against influenza, pneumonia, or both [11].

These rates are comparable with under-vaccination in patients with RA and other autoimmune conditions, ranging from 10% to 35% for influenza vaccine and 17% to 54% for pneumococcal vaccine in some studies [1,2,5,12–14]

The Centers for Disease Control (CDC) identified lack of knowledge about vaccines among adult patients and adult providers as the most substantial barrier to vaccination coverage [15] Several studies demonstrated that vaccination rates among high-risk populations were strongly associated with information received by patients from healthcare providers, underscoring the crucial role physicians have in positively influencing the vaccination behavior of their patients [1,12,16]

Based on these reports, we conducted a retrospective review of historical vaccination rates in our clinics. We used the Carolina Data Warehouse for Health to review the charts of patients with chronic rheumatic disease, as identified by ICD-9 codes for SLE, RA, psoriatic arthritis, polymyositis and dermatomyositis, who were seen at our clinics between 2010 and 2013.

We found that the rates of pneumococcal and influenza vaccination in our patients was approximately 20% and 45% respectively. Limitations include reliance on electronic logging of vaccination data which was not required at the time and usage of limited diagnosis codes as we only reviewed the rheumatology clinic. Despite these limitations, these suboptimal rates of vaccination were in line with national averages and prompted us to explore provider education as an avenue to improve vaccination coverage.

The importance of effective educational activities such as the one proposed here are highlighted by the results of studies demonstrating that vaccination rates among patients with chronic inflammatory conditions were markedly higher among those who had received information about vaccines from their healthcare provider [1–12]

For example, among 485 patients with SLE treated with immunosuppressants, lack of provider recommendation was cited as the reason for failure to receive vaccination in 55% of the 175 patients who did not receive influenza vaccine, and in 87% of the 159 patients who did not receive pneumococcal vaccine [11]

In another example, among 490 patients with rheumatic disease, those who received a recommendation for influenza vaccination by their general practitioner were significantly more likely to be vaccinated than those who did not (57% vs. 15%, adjusted odds ratio [AOR] 6.6, 95% CI 4.1–10.8). This effect was even more significant if they received a recommendation by their rheumatologist (62% vs. 19%, AOR 9.0, 95% CI 4.9–16.5).(12) Yet, only 53.6% of patients claimed to have received information regarding vaccination from one of their healthcare providers [12] In summary, most patients indicate they would get a vaccine on the advice of their physician, and the failure of physicians to recommend vaccination to those at risk is consistently cited as a primary barrier to improved uptake [5,11–13]

Therefore, educational activities that promote healthcare provider awareness about the importance of vaccinations in patients with autoimmune, immunodeficient and allergic respiratory diseases, and educate providers to recognize clinical settings in which vaccines are indicated or contraindicated may increase the rate of appropriate vaccination in these high-risk populations.

## METHODS

### Aim

The aim of this study was to describe prescribers’ perceptions of an educational activity that was developed to increase rates of appropriate pneumococcal and influenza vaccination in adults with chronic inflammatory conditions. We were interested in the impact of the education on the knowledge and practice of providers.

### Study design

An educational activity was developed incorporating concise, multimodal educational approaches (e.g., frequently asked questions, algorithms, and 4 brief case studies) in a single 8-page monograph (Appendix 1). Educational content incorporated Advisory Committee on Immunization Practices guidelines regarding annual seasonal influenza and pneumococcal vaccination [17–19]

The activity was designed to promote awareness of the importance of and outline best practices for pneumococcal and influenza vaccinations in adults with chronic inflammatory conditions, with the ultimate goal being to increase pneumococcal and influenza rates in these patient populations.

This activity was disseminated nationally in online and print formats and presented in a live, interactive “in-house” event at a single site. The presentation slides for the live format were developed from the monograph and this format is comparable to the online/print format.

### Consent

The institutional review board determined that the study was exempt from review, and that individual consent from participants was waived for data collection and/or publishing of de-identified, pooled data that was collected through surveys. Consent for completion of surveys was implied as participants had the option to voluntarily complete the surveys or decline participation. No patients were involved in this activity.

### Participants

In this pilot study, the printed monograph of this educational activity and the digital copy available online (which were approved for continuing medical education credit by the Accreditation Council for Continuing Medical Education [ACCME]) were made available to allergists/immunologists, rheumatologists, fellows, students and other medical professionals *via* the American Academy of Allergy, Asthma & Immunology (AAAAI) website and mailings.

AAAAI members interested in completing the CME activity were able to access the monograph from October 15, 2016 and October 14, 2018 and review the educational activity at their convenience.

Additionally, healthcare providers in our single academic rheumatology, allergy, and immunology division participated in the educational activity in a 1 hour, live, interactive “in-house” event. The information on the monograph was adapted into PowerPoint slides with the addition of the results of the review of historical vaccination rates in our clinics as well as one more case study reflecting the patient population most frequently seen in our clinics.

### Assessments

Participants completed a course evaluation (Appendix 2) to rate their ability to perform the two defined learning objectives for the educational activity upon completion of the activity, and their perception of the quality of the activity and relevance to their practice. Ratings were based on a 5-point Likert scale for both the ability to perform learning objectives (1=not confident, 5=able to demonstrate) and for perceptions of quality and relevance (Excellent to Poor).

Participants were also asked whether they planned to make changes to their practice (patient care, research, or teaching) based on knowledge and skills gained in completing educational activity. In addition, participants completed an online 10-question, multiple-choice and true/false post-test after completing the educational activity *via* print or online. For those participating in the live interactive activity, provider knowledge before and after the activity was assessed *via* a pre-test and post-test utilizing the same 10-question test completed after the online/print activity.

### Statistical analysis

Descriptive statistics were used to assess confidence in vaccination practices and perception of the activity as measured by course evaluation responses. Paired t-test was used to evaluate pre- and post-activity knowledge among the providers who completed a pre- and post-test during the live, interactive format of the activity.

## RESULTS

308 participants completed the activity *via* print or online and took the post-test. The vast majority were physicians, although several physician assistants, nurse practitioners, and nurses completed the activity as well.

The course evaluation was completed by 257 of these participants, which represents an 83% response rate. Among these responders, 93% (n=240) indicated confidence in applying current guidelines for pneumococcal and influenza vaccination and recognizing the clinical settings in which these vaccines are indicated or contraindicated, after completing the activity, defined as a response of ‘4’ or ‘5’ on the Likert scale for ability to perform learning objectives (Figure 1A)

Based on the same Likert scale assessment, 93% (n=240) of respondents indicated confidence in their understanding of the importance of vaccinations in patients with autoimmune disease, including those receiving immunosuppressive therapy, and with chronic inflammatory conditions (Figure 1A).

The majority of respondents perceived the quality of the content (87%, n=223) and format (88%, n=226) of the educational activity to be very good to excellent (Figure 1B) 89% (n=229) of respondents believed the content was very relevant to their practice. 55% (n=142) of respondents planned to change their practice based on knowledge and skills gained in completing the educational activity. Among those who did not plan to change their practice (n=82), 68% felt that their current practice behaviors were reinforced by the activity.

The most common request for further information among respondents was regarding vaccinations in patients undergoing immunoglobulin replacement.

21 participants took part in the live, interactive educational activity, and took the pre- and post-tests assessing knowledge. The mean scores on the pre- and post-tests were 75% (SD 11.6%, 95% CI 70–80%) and 89% (SD 11.1%, 95% CI 85–95%; p=.0001 vs. pre-test score), respectively, demonstrating that knowledge was significantly increased after completing the activity (Figure 1C).

### Learning objective 1

Understand the importance of vaccinations in patients with autoimmune disease, with autoimmune disease on immunosuppressive therapy, and with chronic inflammatory conditions.

### Learning objective 2

Apply the current guidelines for pneumococcal and influenza vaccinations and recognize the clinical settings in which these vaccines are indicated or contraindicated.

## DISCUSSION

The online/print format comprised of a post-test only evaluation of measures and limited our ability to quantify the impact of the educational activity on clinical practice. We used a pre-test/post-test format for the live, interactive format of the activity in order to more specifically assess impact. Additional limitations of our study for all formats of the educational activity include the inability to fully assess potential degree of selection bias given limited baseline characteristic data for participants, and the fact that the knowledge questions were not previously validated.

Clinician education is a strategy previously associated with improvements in influenza and pneumococcal vaccination rates [2,20]. The activity assessed here promotes healthcare provider awareness about the importance of vaccinations in patients with chronic inflammatory diseases and educates providers to recognize clinical settings in which particular vaccines are indicated or contraindicated. It has the flexibility to be accessed in various formats, and should be incorporated into quality improvement endeavors to increase vaccination rates among patients with chronic inflammatory conditions.

## CONCLUSION

In conclusion, this educational activity was effective in increasing clinicians ‘ understanding of the importance of vaccinations in adults with chronic inflammatory conditions. It also improved their confidence in applying the current guidelines for pneumococcal and influenza vaccinations in these high-risk populations, and in recognizing the clinical settings in which these vaccines are indicated or contraindicated.

## Supplementary Material

Suppl doc

## Figures and Tables

**Figure 1A:**
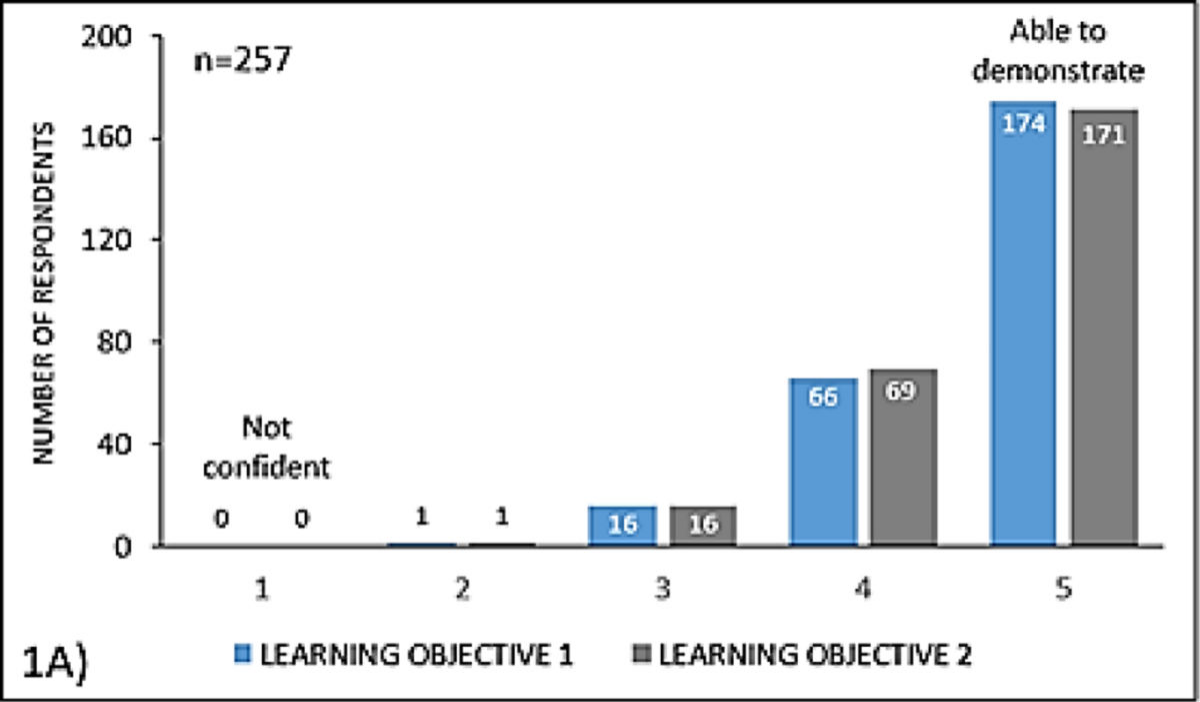
Confidence of participants in their ability to perform learning objectives after completion of the educational activity (print or online)

**Figure 1B:**
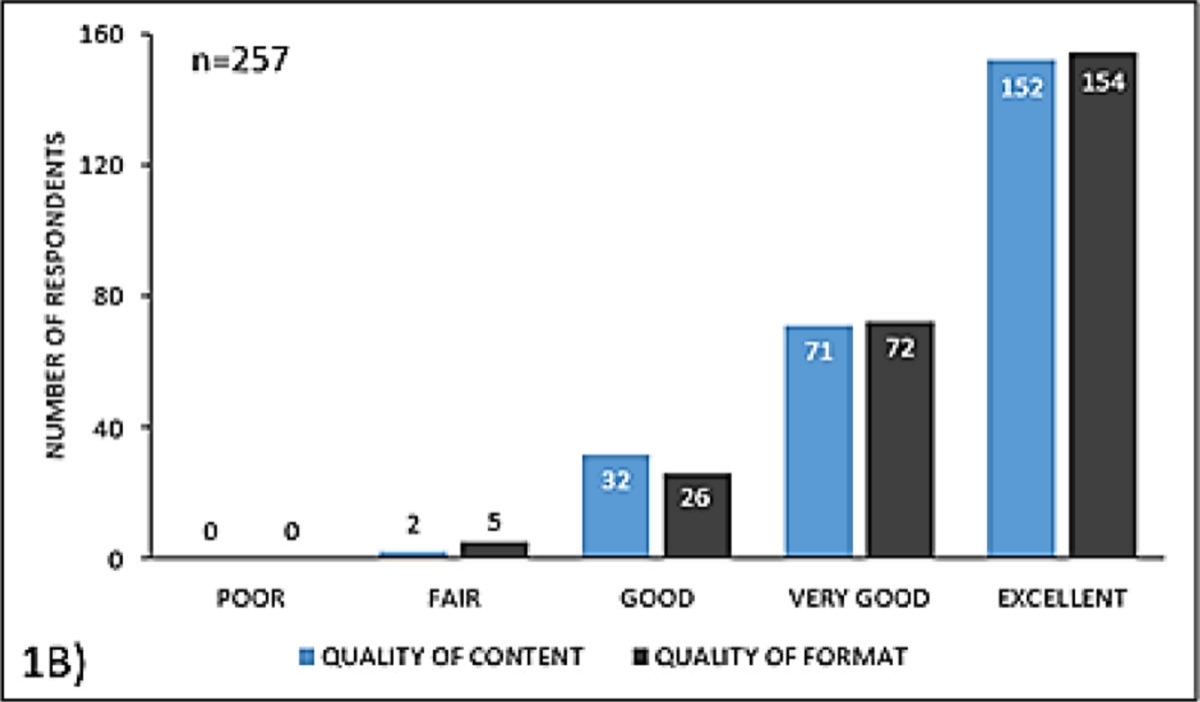
Participant’s perception of the quality of the content and format of the educational activity (print or online)

**Figure 1C:**
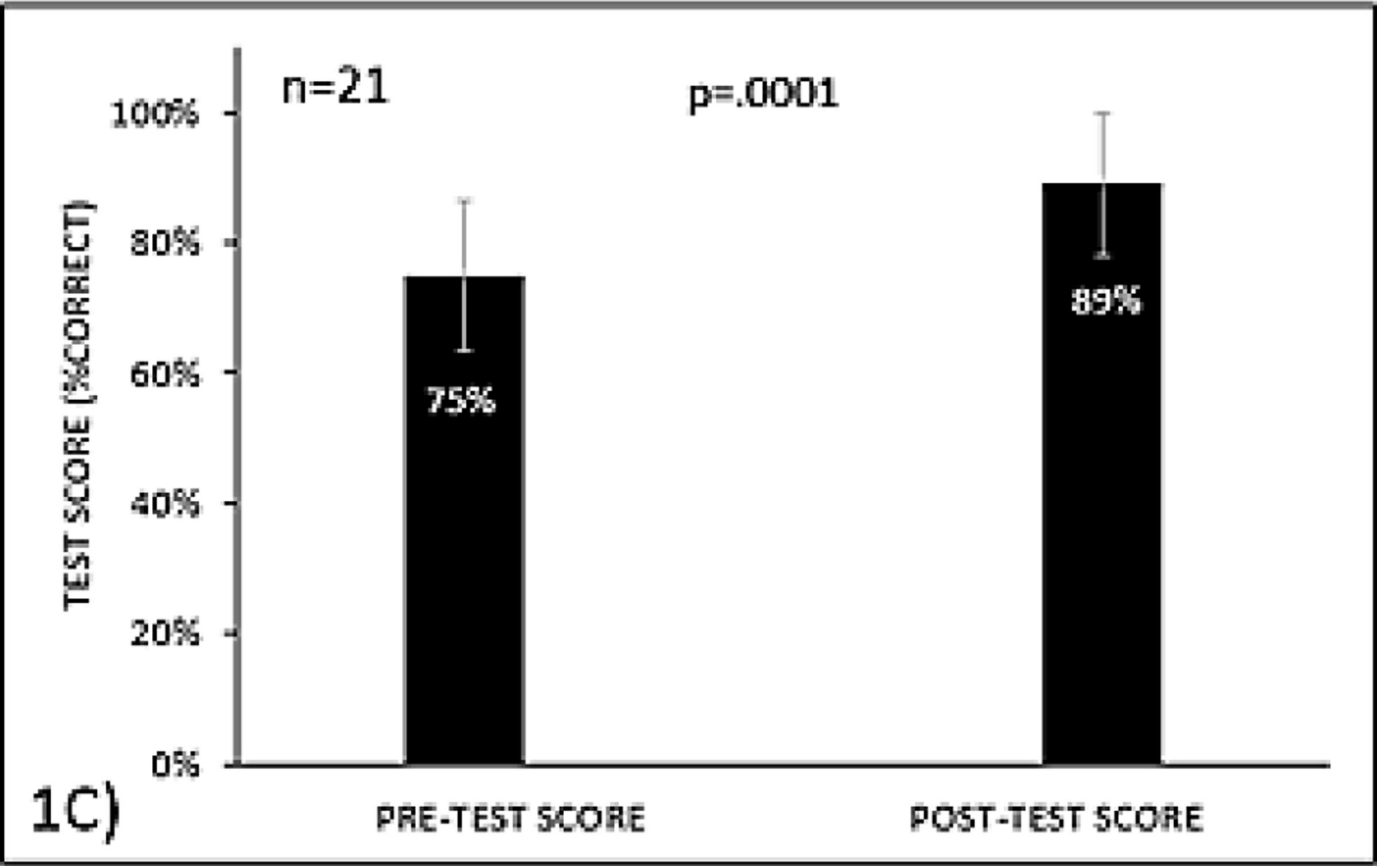
Vaccine knowledge among participants before and after the live, interactive educational activity.
