# Supplementary material for: Clinician’s Perceptions of a CME Activity to Increase Knowledge of Vaccination in Adults with Chronic Inflammatory Conditions
Source: Lupus (Los Angel). Author manuscript; Available in PMC 2020 Feb 12. (PMC7015106; doi:10.35248/2684-1630.19.4.146)
Supplement: Suppl doc [file NIHMS1062269-supplement-Suppl_doc.pdf]

THE UNIVERSITY *of* NORTH CAROLINA *at* CHAPEL HILL
Rheumatology, Allergy & Immunology

Vaccination in Adults with Chronic Inflammatory Conditions

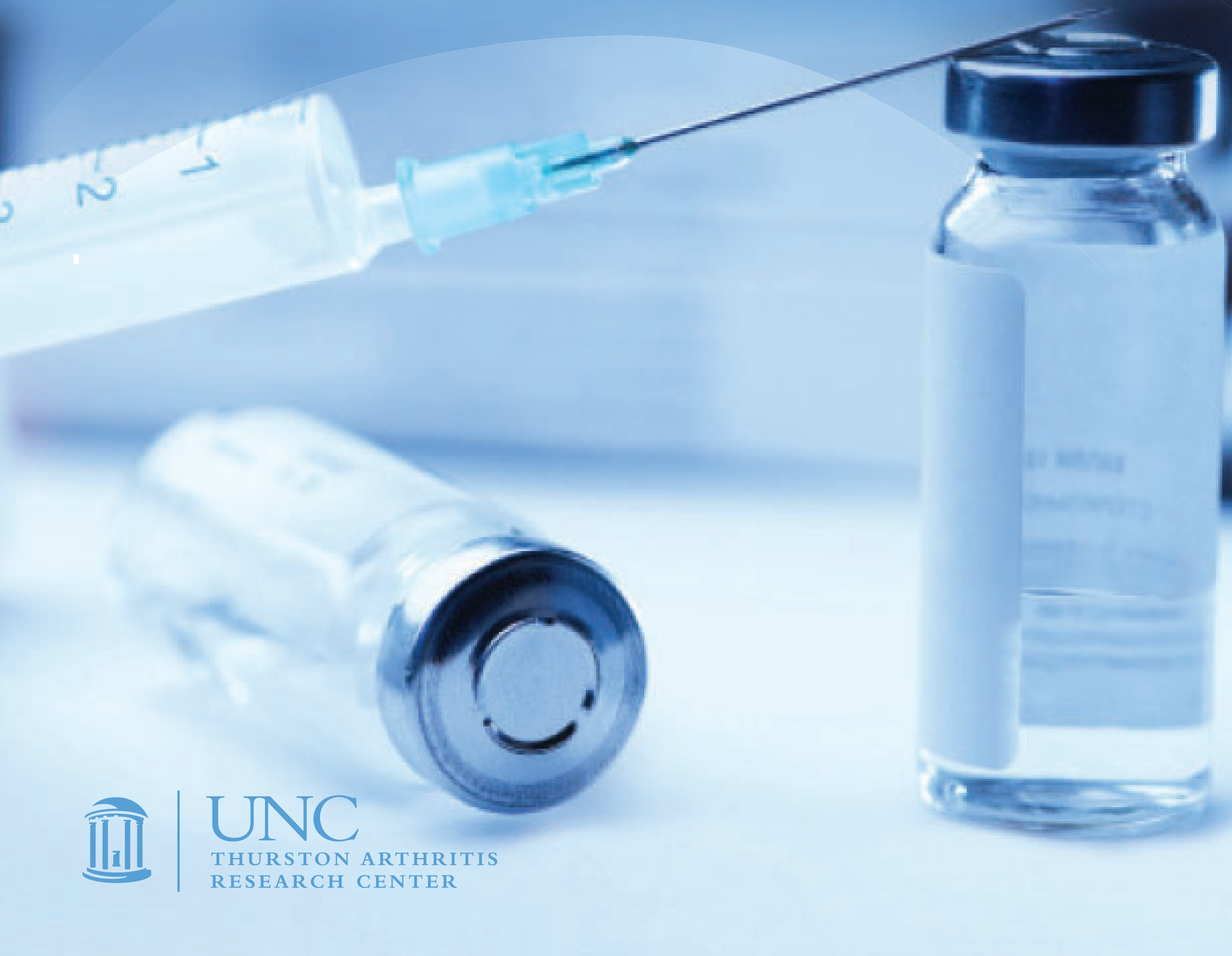

UNC
THURSTON ARTHRITIS RESEARCH CENTER

Overview

Patients with chronic inflammatory diseases are at increased risk for infections. Immunizations are often underutilized in these patients. The objective of this activity is to promote awareness of the importance and outline best practices for pneumococcal and influenza vaccinations in these patient populations.

Target Audience

Vaccination in Adults with Chronic Inflammatory Conditions is intended for allergists/immunologists, rheumatologists, fellows, students and other medical professionals who are interested in learning about vaccination options in patients with autoimmune disease, autoimmune disease on immunosuppressive therapy, primary immunodeficiency and chronic respiratory conditions.

Learning Objectives

The information presented in this educational activity should:

- Promote awareness regarding the importance of vaccinations in patients with chronic inflammatory conditions: autoimmune disease, autoimmune disease on immunosuppressive therapy, primary immunodeficiency and chronic respiratory conditions.
- Allow the participant to be able to apply the current guidelines for pneumococcal and influenza vaccinations and to recognize clinical settings in which these vaccines are indicated or contraindicated.

Accreditation Statement

This activity has been planned and implemented in accordance with accreditation requirements and policies of the Accreditation Council for Continuing Medical Education (ACCME) through the joint providership of the American Academy of Allergy, Asthma and Immunology (AAAAI) and UNC Thurston Arthritis Research Center. The AAAAI is accredited by the ACCME to provide continuing medical education for physicians.

Designation Statement

The American Academy of Allergy, Asthma & Immunology designates this enduring material for a maximum of 1.00 *AMA PRA Category 1 Credit*™. Physicians should claim only the credit commensurate with the extent of their participation in the activity.

AAAAI American Academy of Allergy Asthma & Immunology
www.aaaai.org

Release Date

Vaccination in Adult Autoimmune and Immunodeficiency Conditions was originally released on October 15, 2016.

Expiration Date

This activity will expire on October 14, 2018.

AAAAI Privacy Policy

To verify your participation in online educational activities, you may be asked to provide your name, contact information, and/or other descriptors. The AAAAI will not release this information to outside entities. It may be used internally to inform you of other AAAAI educational activities. If you wish to have your information excluded from this process, please contact us at cme@aaaai.org.

AAAAI Disclosure Policy:

Pursuant to the Code of Ethics for the American Academy of Allergy, Asthma & Immunology (AAAAI) and the Standards for Commercial Support of Continuing Medical Education of the Accreditation Council for Continuing Medical Education, the AAAAI requires disclosure of certain information from faculty members of educational activities designated for CME credit by the AAAAI. Prior to the activity, authors and reviewers are required to disclose all relationships that meet the following parameters:

A. Employment. Name of employer and job title.
B. Financial interests. All organizations, other than the employer, from which the faculty member or a member of his/her immediate family or household receives annual remuneration in any amount (including grants, honoraria and consulting fees).
C. Research interests. All organizations which support research projects for which the faculty member or member of his/her immediate family or household serves as an investigator.
D. Legal Consultation Services/Expert Witness Testimony: All topics on which the faculty member provided legal consultation and/or expert witness testimony during the previous calendar year.
E. Organizational interests. All organizations, other than the AAAAI, for which the faculty member holds volunteer positions.
F. Gifts. All organizations from which the faculty member or a member of his/her immediate family or household have received a gift of any amount in the last year.

G. Other interests. All interests of the faculty member or a member of his/her immediate family or household that would be judged by a majority of his/her peers to be more than casual and/or likely to impact his/her ability to exercise independent judgment. This includes any financial interest in or relationship with any manufacturer of a commercial product, and any financial interest or relationship with any organization that provides commercial support to AAAAI-sponsored educational activities.

The information disclosed by the authors was reviewed in accordance with the AAAAI Disclosure Policy. No relevant financial relationships were disclosed.

This work is funded by an IBM Junior Faculty Development Award and the UNC Thurston Arthritis Research Center

Faculty

Department of Medicine
Division of Rheumatology, Allergy and Immunology
Thurston Arthritis Research Center
The University of North Carolina at Chapel Hill

Biographical Sketches

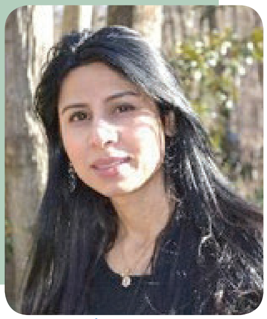

Saira Z. Sheikh, MD

Assistant Professor of Medicine

Dr. Saira Sheikh is board certified in Internal Medicine, Rheumatology and Allergy & Immunology with clinical interests in systemic lupus erythematosus (SLE), allergic rhinitis, urticaria and food allergy. She is director of the UNC Rheumatology Lupus Clinic and the Clinical Trials Program at the UNC Thurston Arthritis Research Center. She is Principal Investigator on several clinical trials for SLE and other rheumatologic conditions, studying new therapies for these diseases. She enjoys teaching and is actively involved in the education of medical students, residents and sub-specialty fellows. Dr. Sheikh is a member of the American Academy of Allergy, Asthma & Immunology, the American College of Allergy, Asthma & Immunology and the American College of Rheumatology.

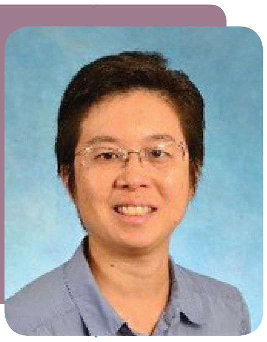

Mildred Kwan, MD, PhD

Assistant Professor of Medicine

Dr. Mildred Kwan is board certified in Internal Medicine and Allergy & Immunology, with clinical interests in chronic urticaria, allergic and non-allergic rhinitis, and asthma. Dr. Kwan's research interests include how immune dysregulation in disease affects the development of atopic and autoimmune conditions, the interaction of the human microbiome on immunological disease, and the effects of biologics and small molecule inhibitors on immune function. Additionally, she has a strong interest in teaching and has developed a teaching curriculum for the Allergy & Immunology fellows at both the University of Pennsylvania and the University of North Carolina. Dr. Kwan is a member of the American Academy of Allergy, Asthma & Immunology.

What We Need to Know About Pneumococcal and Influenza Vaccines

When is the best time to immunize patients with autoimmune disease?

- The best time to immunize patients with autoimmune disease is prior to initiating immunosuppressive therapy.

Can inactivated or killed vaccines be administered once therapy with immunosuppressive agents has been initiated?

- Yes. Inactivated or killed vaccines can be administered during the use of immunosuppressive agents such as disease-modifying antirheumatic drugs (DMARDs), long-term systemic corticosteroids, tumor necrosis factor (TNF) blocking agents and other biological agents.

Are vaccines immunogenic in patients with autoimmune disease?

- Yes. Vaccines remain immunogenic in immunosuppressed patients with autoimmune disease. Several studies have shown that while responses may be diminished compared to disease-free people, patients are able to mount protective antibody titers. [1,2]

Can vaccines increase disease activity in autoimmune rheumatic diseases?

- Overall, the available evidence suggests that vaccines do not increase activity in autoimmune rheumatic diseases such as systemic lupus erythematosus or rheumatoid arthritis. Although disease flares have been reported after vaccination in controlled and uncontrolled studies, [3,4] the frequency of flares was not increased in vaccinated patients compared with unvaccinated patient controls. These flares were therefore believed to represent the natural course of the disease.

  Vaccination is recommended in patients with stable or low disease activity. [5]

Is there any value in immunizing patients with primary immunodeficiency with pneumococcal or influenza vaccines?

- While the true value of immunizing patients with a primary immunodeficiency remains unknown, the standard practice is to follow usual vaccination guidelines for these patients. Injected seasonal influenza, and pneumococcal vaccines should be administered per Centers for Disease Control and Prevention (CDC) guidelines. [6]

Why should we vaccinate against *S. pneumoniae*?

- S. pneumoniae can cause pneumonia, bacteremia and meningitis. There are 400,000 hospitalizations for pneumococcal pneumonia annually and approximately 12,000 cases of pneumococcal bacteremia with a fatality rate of 20%. [7,8,10] Invasive pneumococcal disease causes significant morbidity and mortality particularly in the elderly and in persons with immunocompromising conditions (e.g. primary immunodeficiency, iatrogenic immunosuppression and chronic inflammatory conditions). [10]

Current pneumococcal vaccines approved for use in adults include pneumococcal polysaccharide vaccine-23 (Pneumovax, PPSV23) (1983) and pneumococcal conjugated vaccine-13 (Prevnar, PCV13) (2010).

What are the recent recommendations for the administration of Pneumococcal vaccines?

- With the introduction of PCV13, the rate of Invasive Pneumococcal disease has decreased significantly. This has led to the introduction of new recommendations by the CDC Advisory Committee for Immunization Practices (ACIP) for adults.

For immunosuppressed and immunodeficient patients:

- Pneumococcal vaccine naive:
  - Give PCV13 first.
  - Then give PPSV23 with a minimum of eight weeks from PCV13 administration.
  - Immunodeficient and immunosuppressed patients age 19-64 years receive a second PPSV23 dose five years after the first PPSV23 dose and then a third dose at age 65 years.
- Previously received PPSV23:
  - Give PCV13 at least one year from previous PPSV23 administration.
  - If another dose of PPSV23 is indicated, give at least eight weeks after PCV13 and ≥ five years after initial PPSV23 dose. Immunosuppressed and immunodeficient patients are included in this group.
  - Those who received PPSV23 before age 65 years should receive another dose of the vaccine at age 65 years, or later if at least five years have elapsed since their previous PPSV23 dose.

Chronic Respiratory disease (asthma):

- Asthma patients receive PPSV23 prior to 65 years of age.
- Recommendations for persons ≥ 65 years old are then followed which are:
  - Give PCV13 first.
  - Then give PPSV23 with a minimum of eight weeks from PCV13 administration.

Figure 1: Guidelines for Pneumococcal Vaccines in Immunosuppressed and Immunodeficient Patients

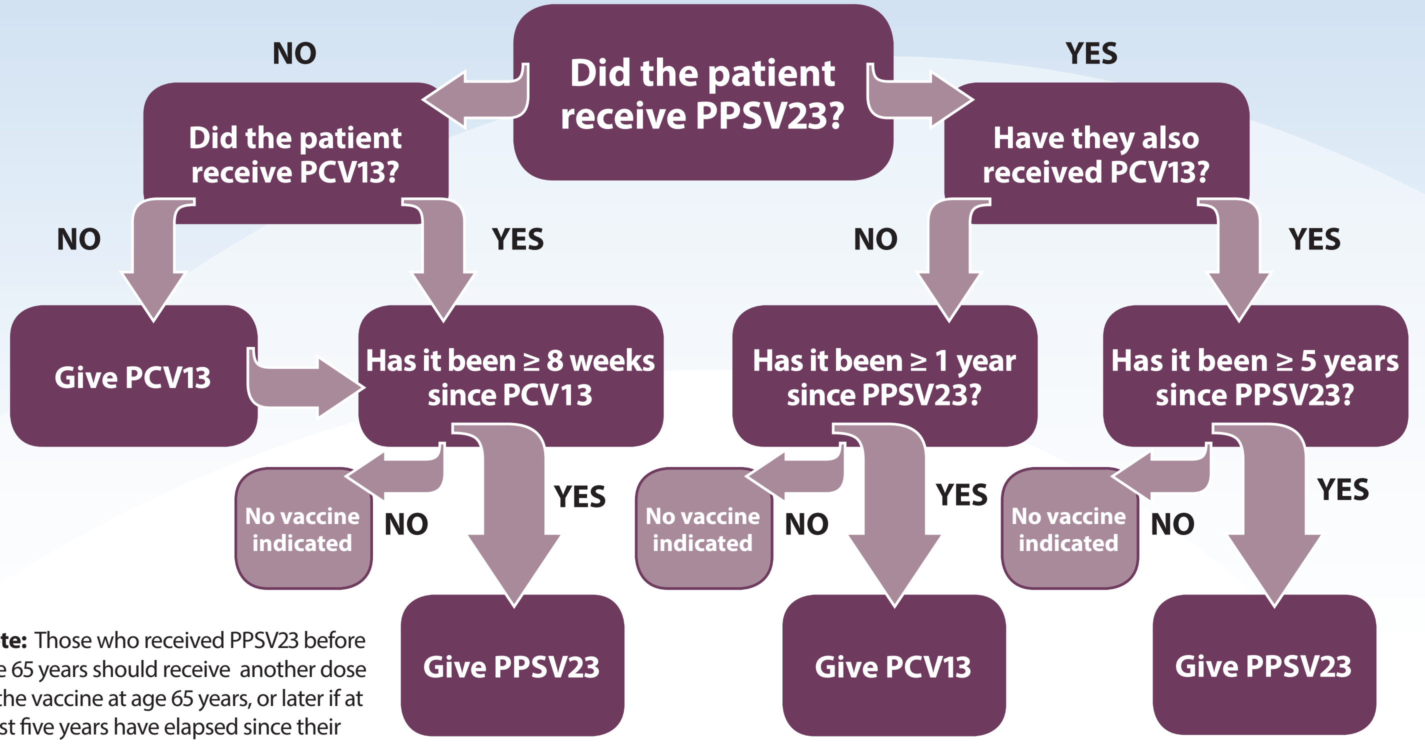

Note: Those who received PPSV23 before age 65 years should receive another dose of the vaccine at age 65 years, or later if at least five years have elapsed since their previous PPSV23 dose.

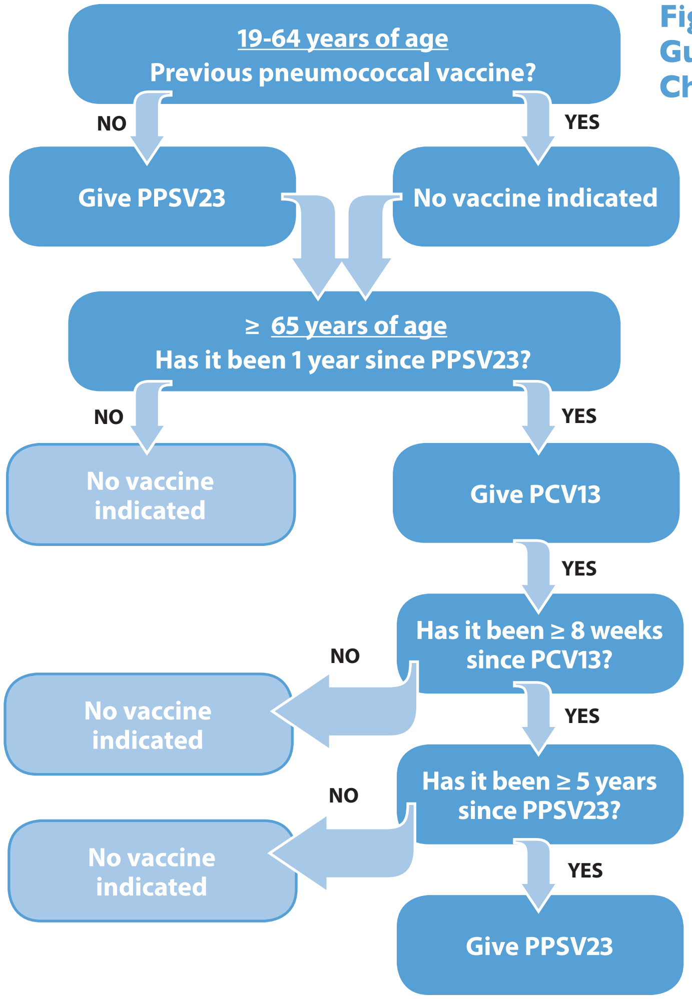

Figure 2: Guidelines for Pneumococcal Vaccines in Chronic Respiratory Disease (Asthma) Patients

Why should we vaccinate against influenza virus?

- Influenza virus causes significant morbidity and mortality with average annual deaths caused by influenza measured at 23,607. 7,9 In the 2009-2010 H1N1 pandemic there were 60 million Americans infected, 270,000 hospitalized and 12,500 deaths.[7,9]

  Persons who are considered at high risk for complications with influenza infection are those with immunodeficiency, on immunosuppressive therapy, chronic respiratory conditions such as asthma and pregnant women.

  Influenza vaccines that are recommended for vaccination are inactivated influenza vaccines (IIVs) which are given by either intramuscular or intradermal routes.

What are the recent recommendations for the administration of influenza vaccine?

- All persons ≥ 6 months should be vaccinated annually.

Case Scenarios for Review:

Case 1: A 32 year old woman has asthma and has not previously received any pneumococcal vaccines. What pneumococcal and influenza vaccines would you consider?

- PPSV23 vaccine now, at age 32
- PCV13 at age 65 followed by a dose of PPSV23 with a minimum of eight weeks from PCV13 administration
- Annual influenza inactivated vaccine

Asthma is not an indication for adult administration of PCV13 < 65 years of age unless the patient has received long-term systemic corticosteroids.

Case 2: A 24 year old woman with common variable immune deficiency received one dose of PPSV23 two years ago. What pneumococcal and influenza vaccines could you consider?

- PCV13 now because one year has passed since patient received PPSV23
- Second dose of PPSV23 at age 27 (≥ 5 years since previous PPSV23 and ≥ 8 weeks since PCV13 dose
- Third dose of PPSV23 at age 65
- Annual influenza inactivated vaccine

Case 3: A 60 year old man with psoriatic arthritis who has not previously received any pneumococcal vaccines and is currently on systemic corticosteriods and will be starting anti-IL-17 therapy. What pneumococcal and influenza vaccines would you consider?

- PCV13 now, at age 60
- PPSV23 at least 8 weeks after the dose of PCV13
- Second dose of PPSV23 at age 65. No further PCV13 doses need to be administered, as the patient received one lifetime dose of PCV13
- Annual influenza inactivated vaccine

Case 4: 24 year old male with ankylosing spondylitis is currently on anti-tumor necrosis factor therapy and asks about getting a flu shot. He reports an "egg allergy" and had hives with egg as a child. Should he receive the influenza vaccine ?

- Inactivated influenza vaccine should be used and administered by clinicians experienced in recognizing and treating anaphylaxis.
- Patient should be observed for 30 minutes in office following the injection.
- Both a single dose or a two step graded challenge are appropriate (10% of the initial dose followed by observation for 30 minutes then the remaining 90% of the dose and a second 30-60 minute observation period).

For reporting adverse events related to vaccinations:

http://vaers.hhs.gov/index

References:

1. Bingham CO 3rd, Looney RJ, Deodhar A, et al. Immunization responses in rheumatoid arthritis patients treated with rituxumab: results from a controlled clinical trial. Arthritis Rheum. 2010;62(1):64-74.
2. van Assen S, Holvast A, Benne CA, et al. Humoral responses after influenza vaccination are severely reduced in patients with rheumatoid arthritis treated with rituximab. *Arthritis Rheum*. 2010;62(1):75-81.
3. Bijl M, Kallenberg CG, van Assen S. Vaccination of immune-compromised patients with autoimmune inflammatory diseases. *Neth J Med*. 2011; 69(1):5-13.
4. van Assen S, Elkayam O, Agmon-Levin N, et al. Vaccination in adult patients with auto-immune inflammatory rheumatic diseases: A systematic literature review for the European League Against Rheumatism evidence-based recommendations for vaccination in adult patients with auto-immune inflammatory rheumatic diseases. *Autoimmun Rev*. 2011;10(6):341-52.
5. van Assen S, Agmon-Levin N, Elkayan O, et al. EULAR recommendations for vaccination in adult patients with autoimmune inflammatory rheumatic diseases. Ann Rheum Dis 2011; 70:414-422.
6. Centers for Disease Control and Prevention - Recommendations and Guidelines: Adult Immunization Schedule. Available at http://www.cdc.gov/vaccines/schedules/index.html
7. CDC Pinkbook (http://www.cdc.gov/vaccines/pubs/pinkbook/index.html),
8. CDC Pneumococcal Vaccination website (http://www.cdc.gov/vaccines/vpd-vac/pneumo/default.htm)
9. CDC Influenza vaccination website (http://www.cdc.gov/flu/professionals/vaccination/index.htm)
10. Human Vaccines & Immunotherapeutics 11:7, 1825—1827 (CAPiTA study).

How to Obtain CME Credit

To receive CME credit for this activity, you must read the entire monograph, register at the American Academy of Allergy, Asthma and Immunology Society (AAAAI) website, complete and pass the post-test and complete the evaluation.

To register on the AAAAI website, please follow these directions.

- Navigate to https://education.aaaai.org/UNCVAC
- Click 'Log in' at top right corner of page
  - For returning users:
    - Click 'I am a member or I have claimed credit through the AAAAI'
    - Enter your AAAAI username and password
      - If you have forgotten your login information, click 'Forgot username or password?'
      - Enter your email address
      - You will receive an email -- follow the instructions to reset your password
      - You should be redirected to the Education site -- if not, after logging in navigate to https;//education.aaaai.org/UNCVAC
  - For new users:
    - Click 'I am not a member and I have never claimed credit through the AAAAI'
    - Enter your email address
      - You will receive an email -- follow the instructions to create a new account (you are not required to join AAAAI to have a user account)
      - You should be redirected to the Education site -- if not, after logging in navigate to https://education.aaaai.org/UNCVAC
- Once you are logged in, click the red begin button in the summary box on the right side of the page
- Read the Instructions page and click the red continue button
- Complete all of the steps, in order, to complete the activity and claim your credits

If you have any questions about the credit process, please feel free to contact the AAAAI Education Staff at cme@aaaai.org or 414-272-6071

AAAAI American Academy of Allergy Asthma & Immunology
www.aaaai.org

UNC
THURSTON ARTHRITIS
RESEARCH CENTER

Thurston Arthritis Research Center
The University of North Carolina at Chapel Hill
3300 Thurston Bldg., CB# 7280
Chapel Hill, NC 27599-7280

Non-Profit
US Postage
PAID
Chapel Hill, NC
Permit No 177

"The most significant barrier to vaccination coverage identified by the CDC is lack of knowledge about vaccines among adult patients and providers ..."

MMWR January 28, 2011 / 60(RR02);1-60

Vaccination Continuing Medical Education (CME) activity course evaluation

1. Please rate your ability to perform the following objectives upon completion of this activity on a scale of 1 to 5, with 1 = Not confident and 5 = Able to demonstrate

   Learning Objective 1: understand the importance of vaccinations in patients with autoimmune disease, with autoimmune disease on immunosuppressive therapy, and with chronic inflammatory conditions

   Learning Objective 2: apply the current guidelines for pneumococcal and influenza vaccinations and to recognize the clinical settings in which these vaccines are indicated or contraindicated

2. Please rate the following (as Excellent, Very Good, Good, Fair, or Poor):
   - Quality of the content
   - Quality of the format
   - Relevance to your practice (patient care, research, teaching)

3. Based on knowledge and skills you gained in completing this activity, do you plan to make changes to your practice (patient care, research, or teaching)?

4. If you plan to change your practice based on what you have learned, please describe below.

5. If you have not made or do not plan to make changes to your practice based on what you learned by completing this activity, please indicate why. Select all that apply:
   - Need to learn more before I feel comfortable changing my practice
   - Knowledge and skills I gained did not have an immediate application
   - Knowledge and skills I gained reinforced my current practice behaviors
   - Financial limitations
   - Staffing limitations
   - Other

6. Did you detect any commercial bias in this activity?

7. If you detected bias, please describe the biased content

8. After completing this activity, what do you need to learn more about to improve your practice (patient care, research, or teaching)?
